# Some Account of a Case of Paruria Inops (Good), or Paralysis of the Kidneys

**Published:** 1830-06

**Authors:** George Hayward

**Affiliations:** Boston.


### PARURIA INOPS.

*Some Account of a Case of Paruria Inops* (GOOD), *or Paralysis of the Kidneys.* By GEORGE HAYWARD, M.D. of Boston.*

THIS disease, in which, according to Dr. Good, the "urine is unsecreted by the kidneys," and there is "no desire to make water, nor sense of fulness in any part of the urinary track," is of very rare occurrence. No writer but Sir Henry Halford, that I am aware of, has published any account of it: this circumstance, together with the fact that its termination is usually, if not always, in death, induces me to submit the following details of a case that recently occurred in my own practice.

On Thursday, July 16th, 1829, at one o'clock P.M. I visited a lady, in the fiftieth year of her age, the mother of several children, who complained of considerable nausea, with diarrhœa and slight pain in the stomach and bowels. She had been as well as usual till Tuesday evening, but since that time had been so much indisposed as to abstain from all food. Her indisposition she attributed to taking cold, from exposure on Monday night. She had formerly been a good deal of an invalid, having suffered severely from repeated miscarriages, but had for the last eight or ten years enjoyed a very tolerable share of health.

I found her tongue covered with an unusually thick coat, her pulse between seventy and seventy-five in a minute, moderately strong, and her skin cooler than in health. I directed a gentle emetic of the wine and powder of ipecacuanha, to be followed by castor oil, and the dejections to be restrained by opium, if they were excessive.

On Friday morning I learnt that the emetic had operated thoroughly but mildly, and that she brought from her stomach food, in an undigested state, that was taken on Tuesday. Her bowels had been so frequently moved as to render it necessary to give her three grains of opium at intervals. She was somewhat stupid, which at the time was attributed to the opium; the coat on the tongue remained

* Ibid.

about the same. She still complained of nausea, though she was free from pain; the pulse was slower than on the preceding day, and the temperature of the skin was diminished. At this visit she told me that she had passed no water since early on Wednesday morning, but that she had no desire to do so, and no pain or inconvenience from it. On passing my hand over the bladder, I satisfied myself that it was not distended: I directed her to take one drachm of a mixture of three parts of the liquid acetate of ammonia, and one part of the spirit of nitrous æther, every two hours, and to let me know in the afternoon if she had not evcacuated the bladder in the interval. I was sent for in the afternoon, as no water had been passed: there was still no suffering, and the bladder was not distended. I then introduced the catheter, and drew off about half an ounce of urine of a very healthy character. The patient was more drowsy at this visit than I had seen her at any previous one, and being now convinced that the whole trouble arose from a want of secretion of urine, I stated to her family that I considered her situation an alarming one, and that the disease would probably have a fatal termination. This surprised them, as her strength was good, she was without pain, and conversed freely, when roused from the stupor to which she was inclined.

I now directed a large blister to be applied over the kidneys, fomentations of hot herbs in spirit above the pubis, sinapisms to the feet, and stimulating frictions to the whole surface of the body, with a continuance of the diuretic mixture.

On Saturday morning all her symptoms were aggravated the pulse slower, the skin colder, and the coma increased. The tongue remained coated, there was no appetite for food, and no water had been passed. A powder, composed of one grain of the submuriate of mercury, five grains of the nitrate of potash, and a scruple of cream of tartar, was ordered to be given every two hours, and the medicine that had been before directed was to be taken in the intermediate hours, and the other remedies were continued. No improvement took place during the day: on the contrary, the coma increased, the pulse became slower and more feeble, and the temperature of the skin was diminished.

Finding all her symptoms worse on Sunday morning, I directed ten drops of the tincture of cantharides and capsicum to be given every two hours, instead of the mixture of the spirits of nitre and Mindererus, and the other remedies to be continued. At this visit I passed the catheter, and drew off about an ounce of healthy urine. At three o'clock

P.M. Dr. Warren saw her with me; she was now so comatose that it was impossible to rouse her, and her pulse had sunk very considerably since morning.

Dr. W. advised to give one drachm of the tincture of cantharides and capsicum every two hours, to rub along the whole course of the spine with the same, and to continue the use of the other means. The medicine was given and the other directions followed till eight o'clock in the evening, when she became unable to swallow, her pulse ceased at the wrist, the surface of the body became cold, and the breathing stertorous, and at long intervals; and in this state she continued till Monday evening, at seven o'clock, when she died.

*Sectio cadaveris, twenty-three hours after death.* The examination was made in presence of my friend, Dr. Homens, of this city.

The general appearance of the body was natural. On dividing the scalp from ear to ear, and dissecting it from the cranium, no fulness was discovered in the vessels of the integuments, and scarcely any blood was effused. The brain and its membranes were found to be in a perfectly healthy state, there was neither effusion nor congestion, but all the appearances warranted the conclusion that the morbid symptoms were owing to the quality of the blood, rather than to its quantity.

There was no mark of disease in the stomach, intestines, liver, spleen, or uterus. The kidney of the right side was about half the usual size, and a third part of it at least was of a deep purple colour, exhibiting traces of considerable inflammation, apparently recent. When cut into, it emitted a strong urinous odour.

The left kidney was not larger than a small English walnut, but of a healthy appearance, and free from any urinous odour. Both the ureters were somewhat inflamed. The bladder did not contain a drop of urine; the mucous coat was nearly black, appearing to have been the seat of violent inflammation. Whether this was the case, or whether the inflammatory appearance about the ureters and bladder was to be attributed to the absence of urine, the usual stimulus of the parts, is a point which I feel unable to decide.

As this disease so rarely occurs, and as all the cases that have come to my knowledge have terminated fatally, I shall be excused perhaps for adding a few remarks. The only printed account of this singular affection which I can find, is the one by Sir Henry Halford, referred to in the beginning of this paper. It was published in 1820, in the 6th volume of the Transactions of the College of Physicians in London. It appears that he had never seen but five cases. They differed in some respects from the one above detailed. "All the patients were fat, corpulent men, between fifty and sixty years of age." "In three of them there was observed a remarkably strong urinous smell in the perspiration twenty-four hours before death." Nothing of this kind was discoverable in my patient.

In Sir H. H.'s patients, no urine whatever was secreted; and he remarks, that "if any water, however small the quantity, had been made in these cases, I should have thought it possible that the patients might have recovered; for it has often surprised me to observe how small has been the measure of that excrementitious fluid which the frame has sometimes thrown off, and yet preserved itself harmless; but the cessation of the excretion altogether is universally a fatal symptom in my experience, being followed by oppression on the brain."

From my patient, it will be recollected, that a small quantity of water was drawn off on Friday afternoon, and again on Sunday morning, showing that some secretion had taken place, which proves that the conjecture in the above quotation, as to the favorable termination of this disease under such circumstances, is unfortunately not to be much relied on.

The disease he denominates paralysis of the kidneys, and till something more is known of it, this name will answer perhaps as well as any other, though if it were fair to draw any conclusion from a single instance, it might be inferred, from the appearances in my case, that the paralysis was consequent on an organic affection. It does not appear that he made any examinations after death, nor has he detailed his method of treatment. Whether this affection is under the control of any remedies we possess, remains to be proved, but hitherto all attempts to check it have been unavailing.

The slow and feeble pulse of my patient, the temperature of her skin, which was below the ordinary standard, and the entire absence of pain, seemed to forbid all depletion, but indicated the administration of stimulants, such especially as would act on the urinary organs. But I must confess that nothing that was administered appeared to have the slightest effect in relieving the patient; and if another case should fall under my care, though I know not what different treatment I could pursue, yet I should feel but little encouragement in adopting my former plan.

Death in these cases is no doubt owing to the impure state of the blood, arising from the failure of the kidneys to perform their usual secretion. The circulating fluid, when it is first received from the lacteals, is in a state wholly unfit to support the vital functions. It is an important part of the office of the lungs, skin, and kidneys, to purify it, and if the customary action of these organs be partially interrupted, alarming consequences ensue, and a complete suspension of their functions produces death. This is well known with regard to the lungs. The immersion of the body into carbonic acid gas is followed by an immediate suspension of vitality, and unless the lungs are soon supplied with respirable air, death is the consequence. The cause of this is, that the pulmonary organs, when deprived of vital air, are unable to effect that peculiar change in the blood which should take place in them; the blood is then sent to the left side of the heart in a state unfit for the purposes of life.

A similar effect, though less sudden, would be produced if there should be a total suspension of the action of the skin, and a failure on the part of the kidneys to perform the office assigned to them, is followed by like consequences. There is a great similarity in the morbid effects arising from these different causes, because the brain is in each case the organ primarily affected. To enable it to perform its functions well, it must be regularly supplied with what is called arterial blood, that is, blood that has been freed of its excrementitious part. But when impure blood is sent to it, it instantly ceases to act if the impurity be great, and immediate death is the consequence. If the noxious principles have been in part removed by the lungs, skin, and kidneys, the effects are not so sudden or violent; coma, however, usually comes on, which gradually increases, if the cause continue, till it terminates in death. When the kidneys, therefore, fail to secrete urine, and thus rid the blood of a part of the excrementitious matter which it contains, the functions of the brain are soon disturbed, and death ensues, unless, as sometimes happens, another organ performs a vicarious office for them.

